# Health warning labels on heated tobacco products and their impact on use intentions and risk perceptions: a cross-sectional study of adult tobacco users in the US and Israel

**DOI:** 10.1186/s13584-023-00582-9

**Published:** 2023-11-13

**Authors:** Zongshuan Duan, Hagai Levine, Yael Bar-Zeev, Yuxian Cui, Cassidy R. LoParco, Yan Wang, Lorien C. Abroms, Amal Khayat, Carla J. Berg

**Affiliations:** 1 Department of Population Health Sciences, School of Public Health, Georgia State University, Atlanta, GA USA; 2 Braun School of Public Health and Community Medicine, Faculty of Medicine, The Hebrew University of Jerusalem and Hadassah Medical Centre, Jerusalem, Israel; 3 Department of Prevention and Community Health, Milken Institute School of Public Health, George Washington University, Washington, DC USA; 4 George Washington Cancer Center, George Washington University, Washington, DC USA

**Keywords:** Heated tobacco products, Risk perceptions, Health communication, Tobacco control

## Abstract

**Background:**

Health warning labels (HWLs) represent an evidence-based tobacco control strategy; however, their application to heated tobacco products (HTPs) and related impacts are understudied. This study examined the impact of HTP HWLs on HTP use intentions and risk perceptions among current tobacco users.

**Methods:**

We analyzed cross-sectional survey data from adults in the US and Israel reporting past-month tobacco use and awareness of HTPs (N = 424). Multivariate analyses examined: (1) sociodemographics in relation to self-reported impact of HTP HWLs (i.e., more concerned about HTP use, reassured, no effect [referent]) among those who noticed HTP HWLs (multinomial regressions); and (2) HWL impacts in relation to HTP use intentions and perceived addictiveness and harm (linear regressions).

**Results:**

Among participants who noticed HTP HWLs (n = 372, 87.7%), 27.7% reported HWLs increased their concerns about HTP use, 22.6% were reassured about use, and 49.7% reported no effect. Factors associated with increased concern (vs. no effect) included other tobacco product use (aOR = 2.10, 95% CI 1.21–3.64) and being female (aOR = 1.77, 95% CI 1.03–3.05). Factors associated with being reassured about HTPs use (vs. no effect) included current HTP use (aOR = 2.11, 95% CI 1.11–4.00) and being from Israel (vs. US: aOR = 3.85, 95% CI 1.85–7.69), female (aOR = 1.91, 95% CI 1.07–3.42), and less educated (< college education: aOR = 2.57, 95% CI 1.42–4.63). Reporting that HWLs on HTPs increased concern (β = 0.46, 95% CI 0.03–0.89) and reassured of use (β = 0.94, 95% CI 0.47–1.41) were positively associated with HTP use intentions; no associations with risk perceptions were found.

**Conclusions:**

Findings indicate that most tobacco users noticed HWLs on HTPs, but the majority reported no effect or being reassured of using HTPs, effects that were magnified for specific subgroups. Both increased concern and reassurance correlated with greater use intentions. Additional research should evaluate HTP HWL impacts and ensure effectiveness in communicating risks and discouraging use.

**Supplementary Information:**

The online version contains supplementary material available at 10.1186/s13584-023-00582-9.

## Background

Over the past decade, the global tobacco market has undergone significant changes with the introduction of products like heated tobacco products (HTPs) [1]. HTPs differ from regular cigarettes, as they heat tobacco to produce an inhalable aerosol instead of combusting it, and differ from e-cigarettes which vaporize an e-liquid (most often containing nicotine) [1, 2]. IQOS, the global HTP leader manufactured by Philip Morris International, was first released in 2014 and has been promoted extensively in over 70 countries [1]. Other major HTP brands include “glo” from British American Tobacco, “Ploom TECH” from Japan Tobacco, “Mok” from China National Tobacco, and “Pulze” from Imperial Brands [3]. While initial research suggests that HTPs might expose users to fewer harmful chemicals than traditional combustible cigarettes [4, 5], the overall public health impact of HTPs remains uncertain [1, 6].

In Israel, IQOS was introduced in 2016 with no regulatory oversight, followed by weak regulation from 2017 to 2018, and then increased regulation including advertising restrictions (since 2019) and plain packaging (since 2020) [7]. In the United States (US), IQOS was launched in October 2019 and expanded across 4 states (i.e., Georgia, North Carolina, South Carolina, Virginia [8, 9]). In July 2020, the US Food and Drug Administration (FDA) authorized IQOS to use messages in its marketing regarding “reduced exposure” to harmful chemicals in comparison to cigarettes, but not referencing “reduced risk” [10]. A patent-infringement lawsuit against a rival company led to the cease of IQOS sales in the US in November 2021 [11]; however, Philip Morris will likely continue promoting the product globally and pursue HTP sales in the US [12, 13].

Health warning labels (HWLs) play a crucial role in informing consumers of the health risks associated with tobacco products in order to discourage use [14]. The most effective HWLs highlight specific and severe health risks like cancer, stroke, and heart disease. Furthermore, pictorial HWLs have been shown to be more effective than textual HWLs, particularly among certain disproportionately-impacted populations [15–17].

Only 6 countries (Canada, Georgia, Israel, Moldova, New Zealand, Ukraine) have specific regulations for HTP HWLs, most commonly required HWLs regarding the product’s health harms and addictiveness [18]. In Israel, all tobacco products, including IQOS, are mandated to display text HWLs that cover 65% of the product packaging (Hebrew on the front and Arabic on the back). There are 13 prescribed warnings, 8 of which reference smoking (e.g., “Medical studies conclude that 85% of all lung cancer cases are due to smoking”), and 5 of which reference cigarettes (e.g., “Cigarettes cause heart disease and stroke”). Additionally, in Israel, plain packaging is obligatory for all tobacco products (except cigars and pipe tobacco sold in specialty shops), which might enhance the visibility of HWLs [3]. In the US, the 2016 FDA deeming rule required all tobacco products to include HWLs on packaging and advertisements beginning in 2018 [19]. HTPs are required to display 2 HWLs that cover at least 30% of the front and back sides of the packaging [19]. These HWLs include one of the 3 rotating Surgeon General's warnings for cigarettes (e.g., “Smoking causes lung cancer, heart disease, emphysema, and may complicate pregnancy”) and an addictiveness statement (i.e., “WARNING: This product contains nicotine. Nicotine is an addictive chemical” [20]). Currently, neither country mandates pictorial HWLs or has HTP-specific HWLs. In the US, tobacco industry litigation has repeatedly delayed the implementation of a 2020 rule requiring pictorial HWLs on cigarette packages [21]. See Additional file 1: Table S1 for an overview of HTP HWL requirements in Israel versus the US.

Recent research indicates that noticing tobacco product HWLs regarding addiction may enhance consumers understanding and recall of health risks [22–24]. Nonetheless, it is also important to study the differential effects of HWLs, as it is plausible that, outside of their anticipated effects (i.e., increasing concern about use), they could have unanticipated effects, such as have no effect or reassuring consumers about use. One longitudinal study found that people who reported that cigarette HWLs increased their concerns about cigarette use were more likely to quit smoking in the future [25]. However, there is limited evidence regarding who notices HWLs on HTPs or their effects on HTP use intentions and risk perceptions. Evidence indicates that these aspects are correlated with HTP use behaviors [26], making it a critical area for research the differential effects of HWLs on HTP use intentions and risk perceptions. Notably, while the International Tobacco Control Project surveys include assessments of the impact of HWLs of e-cigarettes and HTPs including increased concern or reassurance about use [27], little research has examined correlates of reporting reassurance despite some participants reporting being reassured [28, 29]. This is plausible given the marketing of these products as harm reduction products and the possibility of reactance to HWLs, as demonstrated in the literature regarding HWLs on traditional cigarettes [30–33].

Investigating the potential effects of HTP HWLs is especially crucial, considering their ongoing global expansion [34]. It is particularly important to study IQOS, given that it is the HTP market leader globally [35], in Israel (since it was the only HTP sold in Israel until recently [36]), and in the US (until sales were discontinued in 2021 [37]). Furthermore, IQOS’ FDA authorization to use reduced exposure marketing claims in the US adds to the importance of studying this specific product [38]. One concern related to this authorization is that consumers may misunderstand reduced exposure claims to mean reduced risk [38]. Additionally, Philip Morris has used IQOS’ reduced exposure authorization in its advertising in multiple countries [37] and may use messaging attempting to distance IQOS from traditional cigarettes in order to undermine health warnings [39]. Given that HTP HWLs may impact consumers’ intentions to use and risk perceptions [39], it is critical to examine the impacts of HTP HWLs among current and potential HTP users. This study used survey data of US and Israel adults who reported current tobacco use and HTP awareness, and examined: (1) individual factors associated with (a) noticing HWLs on HTPs and (b) perceived effects of HWLs (increased concern of HTP use, reassured use, vs. no effect); and (2) the perceived effects of HWLs in relation to HTP use intentions and risk perceptions.

## Methods

### Data and study sample

This cross-sectional study utilized online survey data collected via Ipsos Panel from October to December 2021 in the US and Israel (detailed elsewhere [40]). Eligibility criteria included: citizen of the respective countries, ages 18–45, English-speaking for US participants, and Hebrew- or Arabic-speaking for Israeli participants. Purposive sampling was used to obtain ~ 40% tobacco users and sufficient representation of racial/ethnic minorities to allow subgroup analyses. The final sample comprised 2222 participants (US n = 1128; Israel n = 1094).

At the beginning of the questionnaire, we stated: “The following questions are aimed at learning more about your perceptions of heated tobacco products. These products heat tobacco but do not actually burn it.” In addition, we displayed images of IQOS devices, device chargers, and heatsticks, without presenting any ads, packaging, or HWLs. We asked participants, “Had you heard of heated tobacco products, like IQOS, which heat sticks of tobacco instead of burning it?” Current analysis was limited to participants who were aware of HTPs and reported currently using cigarettes, e-cigarettes, HTPs, hookah, cigars, pipe, or smokeless tobacco (total n = 424; US n = 125; Israel n = 299). This study was approved by the institutional review boards of George Washington University (NCR213416) and Hebrew University (27062021). Findings are reported following Strengthening the Reporting of Observational Studies in Epidemiology (STROBE) guidelines for cross-sectional studies.

### Measures

All measures were adopted or adapted from the International Tobacco Control Project [27] or the Global Adult Tobacco Survey [41].

*Noticing HWLs and perceived HWL effects.* We adapted measures from the International Tobacco Control Project [27] that assess whether participants notice HTP HWLs and their impact. Participants were asked, “What effect have health warnings had on your thoughts about using heated tobacco products (like IQOS)? have not seen or noticed them, made me concerned about using them, reassured me about using them, had no effect, don't know” [27]. Responses of “don’t know” and “no effect” were collapsed, and 2 variables were created: (1) noticing HWLs: noticed versus had not noticed; and (2) perceived HWL effects among those who noticed HWLs: increased concern, reassured, or had no effect.

*HTP use intentions and risk perceptions.* Use intention was assessed by asking, “How likely are you to try or continue to use HTPs in the next year?” [27, 41]. Perceived harm and addictiveness of HTPs was measured by asking, “How harmful to your health do you think the use of HTPs (such as IQOS) is?” and “How addictive do you think HTPs (such as IQOS) are?” [27, 41]. Response options were 1 = not at all to 7 = extremely.

*Tobacco use.* Four past 30-day tobacco use variables were included in the analysis, including use of HTPs, cigarettes, e-cigarettes, and other tobacco products. Tobacco use was assessed by asking participants to indicate lifetime use of cigarettes, e-cigarettes, HTPs, hookah, cigar products, pipe tobacco, and smokeless tobacco [27, 41]. Among those reporting lifetime use of each product, number of days used in the past 30 days was assessed (any vs. none, for each) [27, 41]. Hookah, cigar, pipe, and smokeless tobacco use was collapsed into a single measure, represented as “other tobacco” use.

*Sociodemographics.* Covariates included age (18–25, 26–35, and 36–45), sex (male, female), sexual orientation (heterosexual, sexual minority), race/ethnicity (in the US: White, Black, Asian, Hispanic; in Israel: Jewish, Arab), and educational attainment (< college degree, ≥ college degree).

### Data analysis

Data management and analyses were conducted using Stata 15.1 (StataCorp). In order to focus on the group of adults for whom HTPs would be most relevant, we analyzed data from participants who reported current use of any tobacco product and being aware of HTPs (N = 424). Descriptive and bivariate analyses were conducted to characterize participants who noticed HTP HWLs versus had not, and who reported different perceived HTP HWL effects. Chi-square tests were used for categorical variables; t-tests or ANOVAs were used for continuous variables.

Multivariable logistic regression analyses were conducted to examine sociodemographic and tobacco use characteristics in relation to: (1) noticing HTP HWLs (binary: yes vs. no) and (2) HTP HWL effects among those who noticed them (multinomial: increased concerns about use vs. no effect, reassured them about use vs. no effect, reassured vs. increased concerns). Multivariable linear regression analyses were conducted to estimate the associations between HWL effects and HTP use intentions and risk perceptions (i.e., addictiveness, harm), controlling for individual sociodemographics and tobacco use status. Country-specific models were also conducted; results were generally similar, so total sample models were presented. Any distinct country-specific model findings are noted as footnotes in the tables. All statistical tests were 2-tailed, and the significance level was set at α = 0.05.

Note that preliminary analyses informed subsequent analytic decisions. First, we examined the distribution of responses across outcomes (i.e., didn’t notice HTP HWLs n = 52 [12.2%] vs. noticed n = 372 [87.8%]; among those who noticed, greater concern n = 103 [27.7%] vs. reassured n = 84 [22.6%] vs. no effect n = 140 [37.6%] vs. don’t know n = 45 [12.1%]). Then, we examined whether and how to include “don’t know” responses to HTP HWL impact assessments. In bivariate analyses, correlates of responding “don’t know” and “no effect” were very similar; furthermore, in multivariable analyses including versus excluding “don’t know” from the “no effect” outcome, findings did not change (except in some cases where significant findings became marginally significant due to reduced power). Thus, we collapsed responses of “don’t know” and “no effect” for multivariable regression analysis. Second, we also examined key stratification variables (e.g., by country, HTP use status), and results were largely the same although some significant results became marginally significant due to power issues. (Also note that there was considerable overlap between HTP use and use of cigarettes and e-cigarettes [40], so the distinct contribution of the HTP variable was minimal.) Thus, we chose to present overall models.

## Results

### Sample characteristics

As shown in Table 1, among the 424 adult tobacco users aware of HTPs (US n = 125; Israel n = 299), 34.7% were female, 24.3% aged 18–25 years, 38.9% aged 26–35 years, 15.3% identified as sexual minorities, and 46.9% had less than a college education. The proportions reporting past 30-day use were: 78.2% cigarette, 56.0% e-cigarette, 24.8% HTP, and 53.5% other tobacco products. Of adult tobacco users aware of HTPs, the majority (87.7%) reported ever noticing HTP HWLs; roughly the same proportion of current cigarette, e-cigarette, and HTP users noticed HTP HWLs (87.5%, 86.1%, and 88.6%, respectively). Of those who noticed HTP HWLs, 27.7% reported the HWLs increased their concerns about use, 22.6% were reassured about HTPs use, and 49.7% were categorized as reporting no effect (including “don’t know” responses). Additional file 1: Table S2 provides country-specific data.

**Table 1:** Bivariate analysis examining factors associated with noticing HTP HWLs among current tobacco users who were aware of HTPs (N = 424) and with HWL effects among those who had noticed HWLs (N = 372)

	Overall	Noticed HTP HWLs		*p*	HWL effect on HTP use (among those who noticed HWLs)			*p*
		No	Yes		Concerned	No effect	Reassured	
	N = 424 (100%)	N = 52 (12.3%)	N = 372 (87.7%)		N = 103 (27.7%)	N = 185 (49.7%)	N = 84 (22.6%)	
	n (%)	n (%)	n (%)		n (%)	n (%)	n (%)	
*Current tobacco use status*								
Cigarettes	329 (78.2)	41 (80.4)	288 (77.8)	.679	76 (74.5)	143 (77.7)	69 (82.1)	.459
No	92 (21.9)	10 (19.6)	82 (22.2)		26 (25.5)	41 (22.3)	15 (17.9)	
E-cigarettes	237 (56.0)	33 (63.5)	204 (55.0)	.249	55 (53.4)	93 (50.5)	56 (66.7)	**.045**
No	186 (44.0)	19 (36.5)	167 (45.0)		48 (46.6)	91 (49.5)	28 (33.3)	
Heated tobacco products	105 (24.8)	12 (23.1)	93 (25.1)	.756	26 (25.2)	37 (20.1)	30 (35.7)	**.024**
No	318 (75.2)	40 (76.9)	278 (74.9)		77 (74.8)	147 (79.9)	54 (64.3)	
Other tobacco*	227 (53.5)	32 (61.5)	195 (52.4)	.217	66 (64.1)	87 (47.0)	42 (50.0)	**.019**
No	197 (46.5)	20 (38.5)	177 (47.6)		37 (35.9)	98 (53.0)	42 (50.0)	
*Demographics*								
Country								
US	125 (29.5)	15 (28.9)	110 (29.6)	.915	32 (31.1)	65 (35.1)	13 (15.5)	**.004**
Israel	299 (70.5)	37 (71.2)	262 (70.4)		71 (68.9)	120 (64.9)	71 (84.5)	
Age								
18–25	103 (24.3)	13 (25.0)	90 (24.2)	.598	26 (25.2)	43 (23.2)	21 (25.0)	.899
26–35	165 (38.9)	23 (44.2)	142 (38.2)		42 (40.8)	68 (36.8)	32 (38.1)	
36–45	156 (36.8)	16 (30.8)	140 (37.6)		35 (34.0)	74 (40.0)	31 (36.9)	
Gender								
Female	147 (34.7)	23 (44.2)	124 (33.3)	.122	38 (36.9)	51 (27.6)	35 (41.7)	.050
Male	277 (65.3)	29 (55.8)	248 (66.7)		65 (63.1)	134 (72.4)	49 (58.3)	
Sexual orientation								
Heterosexual	359 (84.7)	43 (82.7)	316 (85.0)	.673	83 (80.6)	159 (86.0)	74 (88.1)	.312
Sexual minorities	65 (15.3)	9 (17.3)	56 (15.1)		20 (19.4)	26 (14.1)	10 (11.9)	
Educational attainment								
Less than college degree	199 (46.9)	21 (40.4)	178 (47.9)	.312	42 (40.8)	86 (46.5)	50 (59.5)	**.034**
College degree or more	225 (53.1)	31 (59.6)	194 (52.2)		61 (59.2)	99 (53.5)	34 (40.5)	

^*^Other tobacco includes hookah, cigar, pipe, and smokeless tobacco. Bold indicates *p* < .05. Race/ethnicity was not associated with noticing HTP HWLs or effects in the US or Israel

### Factors associated with noticing HTP HWLs and HTP HWL perceived effects

Multivariable binary logistic regression (Table 2) indicated no statistically significant associations between current tobacco use status or sociodemographics and noticing HTP HWLs. Factors associated with reporting greater concern (vs. no effect) included other tobacco product use (aOR = 2.10, 95% CI 1.21–3.64) and being female (aOR = 1.77, 95% CI 1.03–3.05), controlling for other variables. Factors associated with being reassured about HTPs (vs. no effect) included current HTP use (aOR = 2.11, 95% CI 1.11–4.00), being from Israel (vs. US: aOR = 3.85, 95% CI 1.85–7.69), being female (aOR = 1.91, 95% CI 1.07–3.42), and being less educated (aOR = 2.57, 95% CI 1.42–4.63). Factors associated with being reassured about HTPs (vs. greater concern) included current HTP use (aOR = 2.11, 95% CI 1.04–4.27), being from Israel (vs. US: aOR = 3.23, 95% CI 1.45–7.14), and being less educated (aOR = 3.03, 95% CI 1.58–5.83).

**Table 2:** Multivariable logistic regression analyses examining correlates of noticing HTP HWLs among current tobacco users who were aware of HTPs (N = 424) and of HWL effects among those who noticed HWLs (N = 372)

	Noticed HTP HWLs		HWL effect (among those who noticed HWLs)					
			Concerned [Ref: no effect]		Reassured [Ref: no effect]		Reassured [Ref: concerned]	
	aOR	95% CI	aOR	95% CI	aOR	95% CI	aOR	95% CI
*Current tobacco use status*								
Cigarettes (Ref: no)	0.81	0.38, 1.73	0.92	0.51, 1.67	1.07	0.53, 2.17	1.16	0.54, 2.49
E-cigarettes (Ref: no)	0.65	0.35, 1.23	1.04	0.63, 1.72	1.66	0.94, 2.94	1.60	0.85, 3.01
Heated tobacco products (Ref: no)	1.60	0.76, 3.37	1.00	0.54, 1.86	**2.11**	**1.11, 4.00**	**2.11**	**1.04, 4.27**
Other tobacco products* (Ref: no)	0.64	0.34, 1.23	**2.10**	**1.21, 3.64**	1.26	0.69, 2.30	0.60	0.31, 1.18
*Demographics*								
Israel (Ref: US)	1.00	0.50, 2.00	0.83	0.68, 2.13	**3.85**	**1.85, 7.69**	**3.23**	**1.45, 7.14**
Age (Ref: 36–45)								
18–25	0.88	0.38, 2.02	1.06	0.54, 2.10	0.86	0.41, 1.80	0.81	0.35, 1.84
26–35	0.84	0.42, 1.71	1.15	0.65, 2.05	1.05	0.56, 1.98	0.91	0.45, 1.85
Female (Ref: male)	0.60	0.33, 1.12	**1.77**	**1.03, 3.05**	**1.91**	**1.07, 3.42**	1.08	0.57, 2.04
Sexual minorities (Ref: heterosexual)	1.06	0.46, 2.45	1.08	0.54, 2.16	0.57	0.24, 1.34	0.52	0.21, 1.29
< College degree (Ref: ≥ College degree)	1.35	0.72, 2.52	0.85	0.50, 1.44	**2.57**	**1.42, 4.63**	**3.03**	**1.58, 5.83**

Binary logistic regression for noticing HWLs. Multinomial logistic regression for effects (ref: no effect). *Other tobacco includes hookah, cigar, pipe, and smokeless tobacco. Bold indicates *p* < .05. In US-specific models, being Asian (vs. White) was associated with concerned (vs. no effect) and reassured (vs. no effect), but was not included in the table. No other country-specific differences in results in the US- or Israel-specific models

### HTP HWL effects in relation to HTP use intentions and risk perceptions

**Fig. 1:**
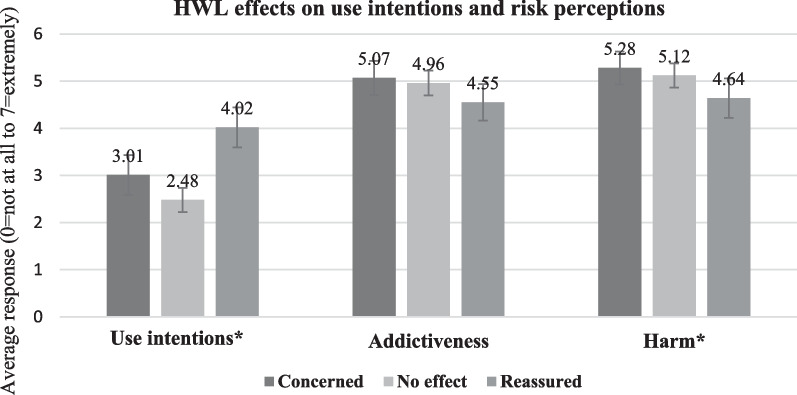
HWL effects on use intentions and risk perceptions. *Notes*: Response options for each assessment: 1 = not at all to 7 = extremely. *ANOVA *p* < .05 for use intentions and perceived harm. Standard errors for use intentions by HWL effect were 0.22, 0.13, 0.22, respectively. Standard errors for perceived addictiveness by HWL effect were 0.18, 0.13, 0.20, respectively. Standard errors for use perceived harm by HWL effect were 0.18, 0.13, 0.21, respectively

As shown in Fig. 1, average use intention was highest among those indicating reassurance by HTP HWLs (Mean = 4.02, SD = 1.98), followed by concern (Mean = 3.01, SD = 2.18) and no effect (Mean = 2.48, SD = 1.77). Average perceived harm was highest among those reporting concern (Mean = 5.28, SD = 1.80) and no effect (Mean = 5.12, SD = 1.77) followed by reassurance (Mean = 4.64, SD = 1.96). There were no significant differences across groups regarding perceived addictiveness.

Multivariable linear regression (Table 3) indicated that, compared to reporting no effect of HTP HWLs, reporting that HTP HWLs increased their concern about HTPs (β = 0.46, 95% CI 0.03, 0.89) or reassured them of HTPs (β = 0.94, 95% CI 0.47, 1.41) were both positively associated with greater use intentions. Additionally, being from Israel (vs. US: β = 0.61, 95% CI 0.21, 1.00), being female (β = 0.55, 95% CI 0.19, 0.92), and reporting current use of cigarettes (β = 0.90, 95% CI 0.48, 1.32), e-cigarettes (β = 1.07, 95% CI 0.72, 1.41), or HTPs (β = 0.97, 95% CI 0.56, 1.38) were associated with greater use intentions.

**Table 3:** Multivariable linear regression analyses examining noticing HWL and their effects in relation to HTP use intentions and risk perceptions among current tobacco users who were aware of HTPs (n = 424)

	Use intentions		Risk perceptions			
			Addictiveness		Harm	
	β	95% CI	β	95% CI	β	95% CI
*HWL effect (Ref: No effect)^*						
Didn't notice	0.04	− 0.51, 0.60	− 0.11	− 0.68, 0.46	− 0.05	− 0.60, 0.50
Concerned	**0.46**	**0.03, 0.89**	0.16	− 0.28, 0.60	0.17	− 0.26, 0.59
Reassured	**0.94**	**0.47, 1.41**	− 0.24	− 0.73, 0.24	− 0.32	− 0.78, 0.14
*Current tobacco use status*						
Cigarettes (Ref: no)	**0.90**	**0.48, 1.32**	**0.45**	**0.02, 0.88**	0.34	− 0.07, 0.75
E-cigarettes (Ref: no)	**1.07**	**0.72, 1.41**	− 0.03	− 0.39, 0.33	− 0.11	− 0.46, 0.23
Heated tobacco products (Ref: no)	**0.97**	**0.56, 1.38**	− **0.90**	− **1.33, **− **0.48**	− **1.09**	− **1.49, **− **0.68**
Other tobacco products* (Ref: no)	0.30	− 0.07, 0.66	− 0.15	− 0.52, 0.22	− 0.10	− 0.46, 0.25
*Demographics*						
Israel (Ref: US)	**0.61**	**0.21, 1.00**	− 0.06	− 0.46, 0.35	0.00	− 0.38, 0.39
Age (Ref: 36–45)						
18–25	− 0.28	− 0.74, 0.19	− **0.57**	− **1.05, **− **0.10**	− 0.31	− 0.77, 0.14
26–35	− 0.32	− 0.71, 0.07	0.01	− 0.39, 0.42	− 0.09	− 0.47, 0.30
Female (Ref: male)	**0.55**	**0.19, 0.92**	0.14	− 0.24, 0.51	0.25	− 0.10, 0.61
Sexual minorities (Ref: heterosexual)	− 0.15	− 0.63, 0.33	− 0.21	− 0.70, 0.29	− **0.60**	− **1.07, **− **0.12**
< College degree (Ref: ≥ college degree)	0.33	− 0.03, 0.69	− 0.33	− 0.69, 0.04	− **0.48**	− **0.83, **− **0.13**

^ Comparing reassured to concerned (ref), reassured was associated with greater use intentions and lower perceived addictiveness and harm. * Other tobacco includes hookah, cigar, pipe, and smokeless tobacco. Boldface indicates *p* < .05. In Israel-specific models, being Arabic (vs. Jewish) was positively correlated with intention to use for HTPs. No other country-specific differences in results in the US- or Israel-specific models

There were no associations between self-reported effects of HTP HWLs and risk perceptions. Current use of cigarettes (β = 0.45, 95% CI 0.02, 0.88) was associated with greater perceived addictiveness, while being 18–25 years old (vs. 36–45: β = − 0.57, 95% CI − 1.05, − 0.10) and current use of HTPs (β = − 0.90, 95% CI − 1.33, − 0.48) were associated with lower perceived addictiveness. Factors associated with lower perceived harm included being a sexual minority (β = − 0.60, 95% CI − 1.07, − 0.12), less than college educated (β = − 0.48, 95% CI − 0.83, − 0.13), and current use of HTPs (β = − 1.09, 95% CI − 1.49, − 0.68).

## Discussion

This study is among the first to investigate the effects of HTP HWLs among adults, specifically tobacco users in a sample comprised of US and Israel adults. In this sample, 87.7% of current tobacco users who were aware of HTPs reported ever noticing HWLs on HTPs, which is higher than the documented rates of noticing HWLs on other tobacco products [28, 42, 43]. For instance, one study using Population Assessment of Tobacco and Health Study (2018–2019) found that about 16% of cigar nonusers and 40–55% of cigar users noticed HWLs on cigar product in the past month [42]. Another study analyzing the 2017 International Tobacco Control Project Youth Tobacco and E-cigarette Survey among 16 to 19-year-olds in Canada, England, and the US found that 33.9% of dual cigarette/e-cigarette users and 21.4% of exclusive e-cigarette users reported noticing e-cigarette health warnings in the past 30 days [43]. Another study using 2016 International Tobacco Control Project data among adults in Australia, Canada, England and the US showed that 15.5% of current cigarette/e-cigarette users reported noticing e-cigarette HWLs in the past month [28]. The high proportion of participants reporting they noticed tobacco product HWLs in our study is likely due to the different measures used (i.e., ever noticing vs. past 30-day noticing) and the nature of the sample analyzed, specifically adults who reported current tobacco use and awareness of HTPs.

Among the most important findings from this study is that roughly equal proportions (~ one-fourth) of participants reported that HTP HWLs increased their concern about HTPs use (27.7%) or reassured them about HTPs use (22.6%), and about half reported no effect of HTP HWLs. Additionally, participants residing in Israel (vs. the US) were more likely to report reassurance from HTP HWLs. Notably, Israel implemented plain packaging and progressive advertising restrictions in 2020, while the US does not have plain packaging requirements and has less comprehensive advertising restrictions (e.g., no point-of-sale display bans). These contextual differences, paired with the longer history and familiarity with IQOS in Israel, may contribute to Israeli participants reporting greater reassurance. Furthermore, self-reported effects of HTP HWLs were not associated with HTP risk perceptions. Together, these findings suggest limited effectiveness of HTP HWLs in informing consumers about HTP-related risks and underscore the need to further examine characteristics of HTP HWLs and the extent to which they might interact with packaging and advertising elements.

Additionally, current HTP users were more likely to be reassured of using HTPs after being exposed to HWLs, which is consistent with literature documenting negative associations between product use and harm perceptions [43, 44]. Notably, HTP ads may target certain groups of people [20]. In this study, females reported greater use intentions and were more likely to report that HTP HWLs reassured them and increased their concern about HTP use (vs. no effect), implying that males had lower use intentions and were more likely than females to report no effect. These findings may reflect some literature suggesting that males pay less attention to messages on health warning labels [45]. In addition, those with less education were more likely to report that HTP HWLs reassured them of HTP use and reported lower HTP risk perceptions, and those who were younger and identified as a sexual minority reported lower HTP risk perceptions. Collectively, these findings highlight the potentially differential impact of HTP marketing on perceptions and use among these groups, the need for prospective research to examine such impacts, and the importance of targeted interventions (e.g., prevention campaigns, cessation efforts) that address the specific factors that may place different groups at high risk for using the various tobacco products (e.g., males).

Interestingly, participants reporting that HTP HWLs increased their concern or reassured them of HTPs both indicated greater use intentions. One possible explanation is the context of the HTP HWLs, particularly the HTP advertising and labeling that surrounds the HWLs [46]. HTP marketing often emphasizes the distinction of HTPs from cigarettes [7, 37], and in the US, the dominant HTP at the time of the survey, IQOS, was authorized to use reduced exposure messaging in its advertising, which was a core message used once authorized [20]. This study found no significant correlations between HTP HWL effects and risk perceptions, similar to previous research examining health warnings for e-cigarettes [47, 48], which may suggest that such HWLs are dismissed or ignored. Additionally, one experimental study found that certain HWL messages, like those promoting “quitting” may be misperceived as only referencing traditional cigarettes [39]. However, HTP HWLs emphasizing disease risk were correlated with increased HTP risk perceptions and lower use intentions [39], suggesting the need for additional research regarding the impacts of different HWL messages.

### Policy implications

Our study findings have important implications for public health practice and policy.

Specifically, we found that half of participants reported no effect of HWLs, while roughly one-fourth reported that HTP HWLs increased their concern about HTPs use or reassured them. Moreover, self-reported effects of HTP HWLs were not associated with HTP risk perceptions, and participants reporting that HTP HWLs increased their concern or reassured them of HTPs both indicated greater use intentions. Finally, certain subpopulations (e.g., females) are distinctly impacted by HTP HWLs. These findings suggest that the current HTP HWLs, which use similar language as those used for combustible cigarette packaging, may create confusion among consumers regarding how such messages should be interpreted (e.g., as applying to HTPs or not [39]). Paired with HTP marketing which often emphasizes their distinction from cigarettes, the lack of HTP HWL specificity may lead consumers to dismiss or misperceive the HWLs [37], and ultimately be reassured about using HTPs. Thus, research is needed to examine how consumers interpret different HWL messages in the context of real-world advertising content and packaging, particularly content using reduced exposure or risk messaging [37]. In addition, further research, including cross-country and experimental studies (e.g., randomized controlled trials), are needed to assess the effectiveness of various types of HTP HWLs (e.g., HWLs on plain packaging, pictorial HWLs, HWL size) on consumers’ perceptions and use behaviors, such as initiating or quitting HTP use, product switching, and poly-product use [16, 24, 49]. Such research should also attend to subpopulation differences, as they may be differentially impacted by specific HWLs [45]. In sum, ongoing research regarding the impact of HTP marketing and HWLs is needed to inform global regulatory efforts, which could include specific regulations requiring HWLs to address the harmful effects of HTP use and/or restricting HTP advertising content (considering its potential to undermine HWLs [39]).

### Limitations

Study limitations include the use of cross-sectional data and self-reported measures, inherently precluding causal inference and introducing potential bias [50]. Findings also have limited generalizability, given that participants were recruited through panels in the US and Israel, and analyses were limited to current tobacco users who were aware of HTPs (done intentionally to ensure findings were relevant to the population examined). Additionally, the inclusion of participants reporting “don’t know” in the “no effect” outcome category may also raise concern [51]; however, preliminary bivariate and multivariable analyses indicated that results excluding versus including “don’t know” responses were similar, with differences likely impacted by reduced power (resulting from excluding 40 participants). Finally, although we controlled for a wide variety of covariates in the analyses, some unknown confounders may not be accounted for (e.g., mental health conditions, other substance use).

## Conclusions

In this sample of US and Israeli adult tobacco users, a high proportion noticed HWLs on HTPs; however, half reported that the HTP HWLs had no effect on them, and roughly equal proportions (about one-fourth) reported that the HTP HWLs made them concerned about HTPs or reassured them about HTPs use. In addition, participants who reported feeling reassured by HWLs were more likely to report an increased intention to use HTPs in the next year, while self-reported HTP HWL effects were not associated with risk perceptions. Moreover, certain subpopulations (i.e., females, young adults, those less educated) may be particularly likely to use HTPs. Findings from this study and from future research building on this work must inform HTP regulatory efforts globally. Such efforts may consider varied approaches to HTP HWLs targeting certain high-risk subpopulations, requiring HWLs to address the harmful effects of HTP use, and/or restricting HTP advertising content, which may have particular implications for the US FDA to reconsider its modified risk tobacco product authorizations.

### Supplementary Information

**Additional file 1. Table S1.** Health warning label (HWL) requirements for all tobacco products, including heated tobacco products (HTPs), in Israel and the US; **Table S2.** Bivariate analysis examining characteristics among current tobacco users who were aware of HTPs from the US and Israel, N = 424.

## Data Availability

The data presented in this study are available on request from the corresponding author. The data are not publicly available due to ethical reasons.

## Abbreviations

aOR: Adjusted odds ratio
CI: Confidence interval
FDA: Food and Drug Administration
HWL: Health warning label
HTP: Heated tobacco product
STROBE: Strengthening the Reporting of Observational Studies in Epidemiology
US: United States

## References

[1] World Health Organization. Tobacco Free Initiative (TFI): heat-not-burn tobacco products information sheet. 2020. https://www.who.int/publications/i/item/WHO-HEP-HPR-2020.2.

[2] National Academies of Sciences, Engineering, and Medicine. Public health consequences of e-cigarettes. 2018. Report No.: 0309468345.

[3] Campaign for Tobacco-Free Kids. Heated tobacco products definition and global market 2020. https://www.tobaccofreekids.org/assets/global/pdfs/en/HTP_definition_en.pdf.

[4] Gale N, McEwan M, Camacho OM, Hardie G, Murphy J, Proctor CJ (2021). Changes in biomarkers of exposure on switching from a conventional cigarette to the glo tobacco heating product: a randomized, controlled ambulatory study. Nicotine Tob Res.

[5] Leigh NJ, Palumbo MN, Marino AM, O’Connor RJ, Goniewicz ML (2018). Tobacco-specific nitrosamines (TSNA) in heated tobacco product IQOS. Tob Control.

[6] Ratajczak A, Jankowski P, Strus P, Feleszko W (2020). Heat not burn tobacco product-a new global trend: impact of heat-not-burn tobacco products on public health, a systematic review. Int J Environ Res Public Health.

[7] Berg CJ, Bar-Zeev Y, Levine H (2020). Informing iQOS regulations in the United States: a synthesis of what we know. SAGE Open.

[8] U.S. Centers for Disease Control and Prevention. Heated tobacco products 2021. https://www.cdc.gov/tobacco/basic_information/heated-tobacco-products/index.html#what-are-htp.

[9] Henderson KC, Van Do V, Wang Y, Duan Z, Popova L, Spears CA (2023). Brief report on IQOS point-of-sale marketing, promotion and pricing in IQOS retail partner stores in Atlanta, Georgia, USA. Tob Control.

[10] U.S. Food & Drug Administration. Modified risk granted orders—exposure modification; 2020. Cited 13 Oct 2022. https://www.fda.gov/media/139797/download.

[11] Corinne Gretler SD. Philip Morris IQOS imports barred from U.S.; deadline passes; 2021. Cited 13 Oct 2022. https://www.bloomberg.com/news/articles/2021-11-29/philip-morris-iqos-imports-barred-from-u-s-as-deadline-passes?leadSource=uverify%20wall.

[12] Gretler C. Philip Morris Plans U.S. IQOS-stick production after import ban: Bloomberg News; 2022. https://www.bloomberg.com/news/articles/2022-02-10/philip-morris-plans-u-s-iqos-stick-production-after-import-ban.

[13] Duan Z, Levine H, Romm KF, Bar-Zeev Y, Abroms LC, Griffith L (2023). IQOS marketing strategies and expenditures in the United States from market entrance in 2019 to withdrawal in 2021. Nicotine Tob Res.

[14] World Health Organization. Framework convention on tobacco control: https://fctc.who.int/who-fctc/overview. Geneva, Switzerland: World Health Organization; 2022.

[15] Hammond D, Reid JL, Driezen P, Boudreau C (2013). Pictorial health warnings on cigarette packs in the United States: an experimental evaluation of the proposed FDA warnings. Nicotine Tob Res.

[16] Noar SM, Hall MG, Francis DB, Ribisl KM, Pepper JK, Brewer NT (2016). Pictorial cigarette pack warnings: a meta-analysis of experimental studies. Tob Control.

[17] O'Connor R (2019). Warnings and packaging. Tob Control.

[18] Tobacco Control Laws. Tobacco Control Laws; 2023. https://www.tobaccocontrollaws.org/legislation/find-by-policy?policy=heated-tobacco-products&matrix=htpMainPolicies&handle=heated-tobacco-products&criteria=health-warnings-on-product-packaging&status=R. Accessed 5 Sept 2023.

[19] US Food and Drug Administration. "Covered" tobacco products and roll-your-own/cigarette tobacco labeling and warning statement requirements; 2020. https://www.fda.gov/tobacco-products/labeling-and-warning-statements-tobacco-products/covered-tobacco-products-and-roll-your-own-cigarette-tobacco-labeling-and-warning-statement. Accessed 5 Sept 2023.

[20] Berg CJ, Romm KF, Bar-Zeev Y, Abroms LC, Klinkhammer K, Wysota CN (2021). IQOS marketing strategies in the USA before and after US FDA modified risk tobacco product authorisation. Tob Control.

[21] U.S. Food and Drug Administration. Cigarette labeling and health warning requirements: https://www.fda.gov/tobacco-products/labeling-and-warning-statements-tobacco-products/cigarette-labeling-and-health-warning-requirements.Washington, DC: US Food and Drug Administration; 2022

[22] Berry C, Burton S (2019). Reduced-risk warnings versus the US FDA-mandated addiction warning: the effects of e-cigarette warning variations on health risk perceptions. Nicotine Tob Res.

[23] Noar SM, Francis DB, Bridges C, Sontag JM, Brewer NT, Ribisl KM (2017). Effects of strengthening cigarette pack warnings on attention and message processing: a systematic review. J Mass Commun Q.

[24] Noar SM, Francis DB, Bridges C, Sontag JM, Ribisl KM, Brewer NT (2016). The impact of strengthening cigarette pack warnings: systematic review of longitudinal observational studies. Soc Sci Med.

[25] Cannoy CN, Bauer SJ, Prakash K, Excell S, Ghosh S, Lundahl LH (2023). Response to health warnings on cigarette packs as a predictor of future smoking among current tobacco smokers. Addict Behav.

[26] Duan Z, Wysota CN, Romm KF, Levine H, Bar-Zeev Y, Choi K (2022). Correlates of perceptions, use, and intention to use heated tobacco products among US young adults in 2020. Nicotine Tob Res.

[27] International Tobacco Control Policy Evaluation Project. International Tobacco Control Policy Evaluation Project: 4-Country Smoking & Vaping W3; 2020. https://itcproject.s3.amazonaws.com/uploads/documents/ITC_4CV3_Recontact-Replenishment_web_Eng_16Sep2020_1016.pdf.

[28] McDermott MS, Li G, McNeill A, Hammond D, Thrasher JF, O'Connor RJ (2019). Exposure to and perceptions of health warning labels on nicotine vaping products: findings from the 2016 International Tobacco Control Four Country Smoking and Vaping Survey. Addiction.

[29] Taylor EV, East KA, McNeill A, Cummings M, Thrasher J, Fong GT (2022). Changes in responses to nicotine vaping product warnings and leaflets in England compared with Canada, the US and Australia: findings from the 2016–2018 ITC Four Country Smoking and Vaping Surveys. Tob Control.

[30] Erceg-Hurn DM, Steed LG (2011). Does exposure to cigarette health warnings elicit psychological reactance in smokers?. J Appl Soc Psychol.

[31] Maynard OM, Attwood A, O'Brien L, Brooks S, Hedge C, Leonards U (2014). Avoidance of cigarette pack health warnings among regular cigarette smokers. Drug Alcohol Depend.

[32] Borland R, Wilson N, Fong GT, Hammond D, Cummings KM, Yong HH (2009). Impact of graphic and text warnings on cigarette packs: findings from four countries over five years. Tob Control.

[33] Emery LF, Romer D, Sheerin KM, Jamieson KH, Peters E (2014). Affective and cognitive mediators of the impact of cigarette warning labels. Nicotine Tob Res.

[34] Abroms L, Levine H, Romm K, Wysota C, Broniatowski D, Bar-Zeev Y (2022). Anticipating IQOS market expansion in the United States. Tob Prev Cessat.

[35] Reuters. Philip Morris beats profit estimates as cost pressures ease amid steady demand; 2023. https://www.reuters.com/business/retail-consumer/philip-morris-beats-quarterly-profit-cost-pressures-ease-amid-steady-demand-2023-07-20/. Accessed 5 Sept 2023.

[36] Khayat A, Berg CJ, Levine H, Rodnay M, Abroms L, Romm KF (2022). PMI’s IQOS and cigarette ads in Israeli media: a content analysis across regulatory periods and target population subgroups. Tob Control.

[37] Berg CJ, Abroms LC, Levine H, Romm KF, Khayat A, Wysota CN (2021). IQOS marketing in the US: the need to study the impact of FDA modified exposure authorization, marketing distribution channels, and potential targeting of consumers. Int J Environ Res Public Health.

[38] Yang B, Massey ZB, Popova L (2021). Effects of modified risk tobacco product claims on consumer comprehension and risk perceptions of IQOS. Tob Control.

[39] Berg CJ, Duan Z, Wang Y, Thrasher JF, Abroms LC, Khayat A (2023). Impact of different health warning label and reduced exposure messages in IQOS ads on perceptions among US and Israeli adults. Prev Med Rep.

[40] Levine H, Duan Z, Bar-Zeev Y, Abroms LC, Khayat A, Tosakoon S (2023). IQOS use and interest by sociodemographic and tobacco behavior characteristics among adults in the US and Israel. Int J Environ Res Public Health.

[41] Global Adult Tobacco Survey Collaborative Group (2020). Global Adult Tobacco Survey (GATS): sample design manual.

[42] Gratale SK, Teotia A, Chen-Sankey J, Ganz O, Delnevo CD, Strasser AA, Wackowski OA (2022). Cigar warning noticing and demographic and usage correlates: analysis from the United States Population Assessment of Tobacco and Health Study, Wave 5. Int J Environ Res Public Health.

[43] Sontag JM, Wackowski OA, Hammond D (2019). Baseline assessment of noticing e-cigarette health warnings among youth and young adults in the United States, Canada and England, and associations with harm perceptions, nicotine awareness and warning recall. Prev Med Rep.

[44] Parker MA, Villanti AC, Quisenberry AJ, Stanton CA, Doogan NJ, Redner R (2018). Tobacco product harm perceptions and new use. Pediatrics.

[45] Kim HK, Chua X (2022). Gender-specific pictorial health warnings: moderation effects of the threat level and gender. J Health Commun.

[46] Liu J, Phua J, Krugman D, Xu L, Nowak G, Popova L (2021). Do young adults attend to health warnings in the first IQOS advertisement in the US? An eye-tracking approach. Nicotine Tob Res.

[47] Mays D, Smith C, Johnson AC, Tercyak KP, Niaura RS (2016). An experimental study of the effects of electronic cigarette warnings on young adult nonsmokers’ perceptions and behavioral intentions. Tob Induc Dis.

[48] Wackowski OA, Sontag JM, Hammond D, O’connor RJ, Ohman-Strickland PA, Strasser AA (2019). The impact of E-cigarette warnings, warning themes and inclusion of relative harm statements on young adults’ E-cigarette perceptions and use intentions. Int J Environ Res Public Health.

[49] Hall MG, Sheeran P, Noar SM, Ribisl KM, Boynton MH, Brewer NT (2017). A brief measure of reactance to health warnings. J Behav Med.

[50] Coughlin SS (1990). Recall bias in epidemiologic studies. J Clin Epidemiol.

[51] Waters EA, Kiviniemi MT, Hay JL, Orom H (2022). Dismissing, “Don’t Know” responses to perceived risk survey items threatens the validity of theoretical and empirical behavior-change research. Perspect Psychol Sci.

