# Supplementary material for: Health warning labels on heated tobacco products and their impact on use intentions and risk perceptions: a cross-sectional study of adult tobacco users in the US and Israel
Source: Isr J Health Policy Res. 2023 Nov 13;12:33. doi: 10.1186/s13584-023-00582-9 (PMC10644544; doi:10.1186/s13584-023-00582-9)
Supplement: Supplementary file 1 — Additional file 1. Table S1. Health warning label (HWL) requirements for all tobacco products, including heated tobacco products (HTPs), in Israel and the US; Table S2. Bivariate analysis examining characteristics among current tobacco users who were aware of HTPs from the US and Israel, N = 424. [file 13584_2023_582_MOESM1_ESM.docx]

| **Supplementary Table 1. Health warning label (HWL) requirements for all tobacco products, including heated tobacco products (HTPs), in Israel* and the US** | | |
| --- | --- | --- |
| ***Country*** | **Israel** | **US** |
| ***Text vs. pictorial*** | Text | Text |
| ***Number of warnings*** | 2 (front: Hebrew; back: Arabic) | 2 |
| ***Size and placement*** | Cover ≥65% of the front and back sides of the packaging | Cover ≥30% of the front and back sides of the packaging |
| ***Content*** | Must be 1 of 13 prescribed warnings, 8 of which reference smoking (e.g., “Medical studies conclude that 85% of all lung cancer cases are due to smoking”), and 5 of which reference cigarettes (e.g., “Cigarettes cause heart disease and stroke”). | One warning must be 1 of the 3 rotating Surgeon General's warnings for cigarettes (e.g., “Smoking causes lung cancer, heart disease, emphysema, and may complicate pregnancy”).  One must be: “WARNING: This product contains nicotine. Nicotine is an addictive chemical.” |
| Notes: * Requirements for electronic cigarettes differ. | | |

| **Supplementary Table 2. Bivariate analysis examining characteristics among current tobacco users who were aware of HTPs from the US and Israel, N=424** | | | | |
| --- | --- | --- | --- | --- |
|  | **Overall** | **US** | **Israel** |  |
|  | **N=424 (100%)** | **N=125 (29.5%)** | **N=299 (70.5%)** |  |
|  | **n (%)** | **n (%)** | **n (%)** | **p** |
| **Noticed HTP HWLs** |  |  |  |  |
| No | 52 (12.3) | 15 (12.0) | 37 (12.4) | .915 |
| Yes | 372 (87.7) | 110 (88.0) | 262 (87.6) |  |
| **HWL effect on HTP use** |  |  |  |  |
| Concerned | 103 (27.7) | 32 (29.1) | 71 (27.1) | **.004** |
| No effect | 185 (49.7) | 65 (59.1) | 120 (45.8) |  |
| Reassured | 84 (22.6) | 13 (11.8) | 71 (27.1) |  |
| **HTP use intentions [M (SD)]** | 2.95 (2.03) | 2.34 (1.83) | 3.20 (2.06) | **<.001** |
| **HTP risk perceptions [M (SD)]** |  |  |  |  |
| Addictiveness | 4.88 (1.85) | 4.98 (1.94) | 4.84 (1.82) | .764 |
| Harm | 5.06 (1.82) | 5.11 (1.65) | 5.04 (1.88) | .628 |
| **Current tobacco use status** |  |  |  |  |
| Cigarettes | 329 (78.2) | 90 (73.2) | 239 (80.2) | .112 |
| No | 92 (21.9) | 33 (26.8) | 59 (19.8) |  |
| E-cigarettes | 237 (56.0) | 64 (51.6) | 173 (57.9) | .239 |
| No | 186 (44.0) | 60 (48.4) | 126 (42.1) |  |
| Heated tobacco products | 105 (24.8) | 24 (19.2) | 81 (27.2) | .083 |
| No | 318 (75.2) | 101 (80.8) | 217 (72.8) |  |
| Other tobacco* | 227 (53.5) | 68 (54.4) | 159 (53.2) | .818 |
| No | 197 (46.5) | 57 (45.6) | 140 (46.8) |  |
| **Demographics** |  |  |  |  |
| Age |  |  |  |  |
| 18-25 | 103 (24.3) | 19 (15.2) | 84 (28.1) | **.010** |
| 26-35 | 165 (38.9) | 50 (40.0) | 115 (38.5) |  |
| 36-45 | 156 (36.8) | 56 (44.8) | 100 (33.4) |  |
| Gender |  |  |  |  |
| Female | 147 (34.7) | 44 (35.2) | 103 (34.5) | .882 |
| Male | 277 (65.3) | 81 (64.8) | 196 (65.6) |  |
| Sexual orientation |  |  |  |  |
| Heterosexual | 359 (84.7) | 106 (84.8) | 253 (84.6) | .962 |
| Sexual minorities | 65 (15.3) | 19 (15.2) | 46 (15.4) |  |
| Educational attainment |  |  |  |  |
| Less than college degree | 199 (46.9) | 78 (62.4) | 121 (40.5) | **<.001** |
| College degree or more | 225 (53.1) | 47 (37.6) | 178 (59.5) |  |
| Notes: *Other tobacco includes hookah, cigar, pipe, and smokeless tobacco. Response options for HTP use intentions and risk perceptions: 1=not at all to 7= extremely. Bold indicates p<.05. Race/ethnicity was not associated with noticing HTP HWLs or effects in the US or Israel. | | | | |
